# 

**Published:** 1842-04

**Authors:** 


### CONTENTS OF No. XXVI.

### PART FIRST.—Analytical and Critical Reviews.

PAGE

ART. I.—1. Histoire Statistique et Morale des Enfants Trouvés, suivie de cent Tableaux. Par J. F. TEOME et J. B. MONFALCON . . . 289
2. Nouvelles Considérations sur les EnfantsTrouvés. Par MM.TEOME et MONFALCON ib.
3. Des Hospices d'Enfants Trouvés en Europe, etc. Par B. B. REMACLE . ib.
4. Recherches sur les Enfants Trouvés, les Enfants Naturels, et les Orphelins en France, etc. Par L'Abbé A. H. GAILLARD . . . ib.

ART. II.—1. De Organische Gebreken der Urethra, beschreven door D. J. A. ARNTZENIUS . . . . . . . 312
The Organic Diseases of the Urethra, described by Dr. J. A. ARNTZENIUS.
2. A Treatise on Stricture of the Urethra, containing an account of Improved Methods of Treatment; with an Appendix on Dilatation by Fluid Pressure in the Treatment of Urinary Calculus and other Diseases. By Dr. ARNOTT . ib.
3. Practical Observations on the Pathology and treatment of Stricture of the Urethra, with Cases. By ROBERT WADE . . . . ib.
4. Observations sur les Rétrécissements de l'Urètre par cause traumatique. Par J. FRANC . . . . . . . ib.
5. On the Treatment of Stricture of the Urethra. By BRANSBY COOPER, F.R.S. ib.

ART. III.—Die Specielle Pathologie und Therapie vom Klinischen Standpuncte ausbearbeitet. Von Dr. CARL CANSTATT . . . . 327
Special Pathology and Therapeutics, founded on Clinical Observation. By Dr. CHARLES CANSTATT.

ART. IV.—1. The Influence of Tropical Climates on European Constitutions. By JAMES JOHNSON, M.D., and RANALD MARTIN, Esq. . . 347
2. Researches into the Causes, Nature, and Treatment of the more prevalent Diseases of India and of warm climates generally. By J. ANNESLEY, F.R.S., F.S.A. ib.
3. The Madras Quarterly Medical Journal. Edited by SAMUEL ROGERS . ib.
4. Transactions of the Medical and Physical Society of Bombay for the year 1840. ib.
5. The Indian Journal of Medical and Physical Science. Edit. by F. CORBYN . ib.

ART. V.—1. De l'Opération de l'Empyème. Par M. C. SEDILLOT . . 364
2. On the Treatment of Chronic Pleurisy with Effusion. By the late Dr. J. HOPE ib.
3. The Article "Empyema," in Cyclop. of Pract. Surgery. By Dr. WAISHE . ib.

ART. VI.—1. The Nature, Causes, and Treatment of Erysipelas. By T. NUNNELEY 373

2. Article, Erysipelas, in Cyclop. of Pract. Surgery. By J. C. DONELLAN, M.D. . ib.

ART. VII.—Traité Pratique de la Pneumonie aux différens ages et dans ses rapports avec les autres maladies aigues et chroniques. Par A. GRISOLLE, D.M.P. . 382

ART. VIII.—Madness, or the Maniac's Hall; a Poem, in Seven Cantos . . 412

ART. IX.—Essai sur les Effets produits dans le Corps Humain par la Luxation congénitale et accidentelle non réduite du Fémur. Par G. VROLIK, M.D. . 420

ART. X.—Statistical Researches relative to the Etiology of Pulmonary and Rheumatic Diseases. By SAMUEL FORRY, M.D. . . . 423

ART. XI.—Séméiotique des Urines, ou Traité des altérations de l'Urine dans les Maladies; suivi d'un Traité de la Maladie de Bright aux divers âges de la vie. Par ALF. BECQUEREL, M.D. . . . . . 435

ART. XII.—On Rheumatism, in its various forms, and on the Affections of Internal Organs, more especially the Heart and Brain, to which it gives rise. By RODERICK MACLEOD, M.D. . . . . . 445

ART. XIII.—Bidrag til Kundskab af de endemiske Hudsygdomme. Af Dr. HJORT 461

Contributions to the knowledge of Diseases of the Skin. By Dr. HJORT.

ART. XIV.—Principles of Human Physiology, with their chief applications to Pathology, Hygiène, and Forensic Medicine; especially designed for the use of Students. By WILLIAM B. CARPENTER, M.D. . . . 467

ART. XV.—1. Compte Rendu des Séances de l'Académie des Sciences, No. V.—Août, 1841. (Rapport fait à l'Académie des Sciences, au nom de la Commission dite de la Gélatine) . . . . 486

§

PAGE

2. Proceedings of the Philosophical Society of Glasgow. (Report "On the best means of providing cheap and nourishing food for the Poor.") . . 486

Art. XVI.—On the Remote Cause of Epidemic Disease. By John Parkin . 497

Art. XVII.—Verhandelingen van het Genootschap ter Bevordering der Genees en Heelkunde te Amsterdam . . . . . . 503
Transactions of the Society for the promotion of Medicine and Surgery at Amsterdam.

Art. XVIII.—1. Advice to the Deaf, &c. By John Harrison Curtis, Esq. . 509
2. The Cephaloscope, and its uses, &c. By J. H. Curtis, Esq. . . ib.
3. Deafness successfully treated, &c. By J. Yearsley, M.R.C.S. . . ib.
4. Improved Methods of treating Diseases of the Ear. By J. Yearsley, M.R.C.S. ib.
5. An Outline of the principal Diseases of the Ear, &c. By J. Yearsley, &c. . ib.
6. On Deafness, &c. By J. Yearsley, &c. . . . . ib.

### Part Second.—Bibliographical Notices.

Art. I.—Report of the Poor Law Commissioners on Medical Charities in Ireland. 513

Art. II.—Dissertation on the Perforating Ulcer of the Stomach, &c. By Dahlerup 516

Art. III.—On the Structure, Economy, and Diseases of the Ear. By G. Pilcher 517

Art. IV.—A Practical Treatise on Auscultation. By MM. Barth and Roger . 518

Art. V.—Nouvelle Manuel d'Anatomie. Par Dr. Crommelinck. . . 519

Art. VI.—The Means of Promoting and Preserving Health. By T. Hodgkin, M.D. ib.

Art. VII.—1. An Exposition of the Pathology of Hysteria. By Dr. E. O. Hocken. 520

2. An Essay on the Influence of Constitution. By Edward Octavius Hocken, M.D. ib.

Art. VIII.—The Retrospective Address delivered at the Ninth Anniversary Meeting of the Provincial Medical and Surgical Association. By R.J.N. Streeten, M.D. ib.

Art. IX.—Elements of Materia Medica and Pharmacy. By O'Bryan Bellingham, M.D., &c. Edited by Arthur Mitchell, M.D. . . . 521

Art. X.—Natural History of Man. By J. C. Prichard, M.D., F.R.S., M.R.I.A. ib.

Art. XI.—Observations on the Analogy between Ophthalmic and other Diseases, being the substance of a Paper read at King's College, London. By C. Vines. 522

Art. XII.—A Dispensatory, or Commentary on the Pharmacopœias of Great Britain; comprising the Natural History, Description, Chemistry, Pharmacy, Actions, Uses, and Doses of the Articles of the Materia Medica. By Robert Christison, M.D. F.R.S.E. . . . . ib.

### Part Third.—Selections from the British and Foreign Journals.

### I. THE FOREIGN JOURNALS.

#### ANATOMY AND PHYSIOLOGY.

PAGE

Dr. Stilling on the Formation of Contagious Confervæ on Living Frogs . 523
Dr. G. Simon on the Development of the Hair . . . . 525
Dr. A. Numan on a Description of a hitherto-undescribed Entozoon . . 526
M. Bonafont on the Movements of the Bones of the Internal Ear . . ib.
Dr. M. Payan on a complete Anchylosis of the Temporo-Maxillary Articulations 527
Dr. Friese on a Remarkable Monstrosity . . . . ib.
M. Voisin's Account of a Phrenological Visit to the Penitentiary at Paris . ib.

#### PATHOLOGY, PRACTICAL MEDICINE, AND THERAPEUTICS.

Dr. Levy on Inflam. of Umbilical Arteries, as a cause of Trismus Neonatorum 528
Prof. de Renzi on an Epidemic of Typhus in Cervaro in 1840 . . 529
Prof. Eschricht on the Diceras, a rare form of intestinal worm . . 532

PAGE
Dr. Kanzler on a Disease of Sappers and Miners . . . 532
Dr. Scharlau on the Contagious Nature of Pemphigus Neonatorum . 533
MM. Rayer and Bricheteau's Account of an Epidemic of Sweating Sickness, &c. 534
M. Durand Fardel's Memoir on Softening of the Brain . . 535
M. de Castelnau's Researches into the Physical Causes of Metallic Tinkling, &c. 536
Dr. S. F. Simon's Analysis of the Urine in Scarlatina . . . 537
M. Bricheteau's Researches into the real Nature of Œdema of the Glottis . ib.
M. Bouillaud's Account of a case of Acute Glanders in the Human Subject . 538
Dr. Bouchardat's Researches on Saccharine Diabetes . . . 539
Prof. Hoppe on Larvæ of Insects (Musca Stabulans) passed by Stool . 540
Prof. Bang on Typhus in the Royal Frederic Hospital at Copenhagen . ib.
MM. Solon on Oxide of Zinc in Eczema, &c. . . . . 541

SURGERY.

M. Lasalle on Rupture of the Spine, by a violent muscular effort . . 542
Dr. Casper on a Wound through the Sternum and the Arch of the Aorta . ib.
Dr. Schlesier on the Treatment of Ulcers between the toes . . ib.
On Syphilitic muscular Retraction, and its Treatment . . . 543
M. Lisfranc on the means of arresting Hemorrhage . . . ib.
M. Velpeau on Iodine Injections in the Treatment of Serous Cysts . . ib.

MIDWIFERY, AND DISEASES OF WOMEN AND CHILDREN.

MM. Barthez and Rilliet on Chronic Hydrocephalus in a Boy 9 years of age ib.
MM. Mathieu and Bressolles on a Case of Extra-Uterine Pregnancy, &c. . 545
M. A. Robert on Inflammation of the Mucous Follicles of the Vulva, &c. . 546
Prof. Trousseau on the Pulse of Infants at the Breast . . . 547
Dr. Stilling on the Extirpation of a Diseased Ovary . . . ib.
Dr. Schöller on Injury to the Head of a Child in its passage through the Pelvis 548
Dr. Borggreve on Spontaneous Inversion and gradual Replacement of the Uterus ib.
Dr. Dommes on the employment of a Ring in Prolapsus Uteri . . 549
Dr. Marotte on Treatment of Croup . . . . . ib.

ANIMAL CHEMISTRY AND PHARMACY.

M. F. Boudet's Remarks on the Memoir of Dr. Dupasquier, &c. . . 550

MEDICAL STATISTICS.

Dr. Lohmeyer on Results of Revaccination in the Prussian Army in 1840 . 550
Dr. Muhry on Results of Revaccination in the Hanoverian Army in 1837-8-9 . 551
Statistics of Suicide in France . . . . . 552

---

### II. THE BRITISH JOURNALS, &c.

ANATOMY AND PHYSIOLOGY.

Dr. Zabriskie's Case of Paralysis of the Portio Dura, &c. . . . 553
Mr. Bowman on Minute Anatomy of Fatty Degeneration of the Liver . . ib.
Dr. Barry on Fibre. . . . . . . 554
Asymmetrical Development. . . . . . 557

PATHOLOGY, PRACTICAL MEDICINE, AND THERAPEUTICS.

Dr. James Johnson on Partial Hypertrophy of the Heart . . . 557
Dr. Graves on Abscesses of the Lungs . . . . . ib.
Dr. Bird on Pyrosis from an elongated and Depressed Ensiform Cartilage . 558
Mr. Erichsen on the Seat and Nature of Favus . . . ib.
Dr. Crawford on Bloodletting in Mania . . . . 559
Mr. John Paterson on Vaccine Virus . . . . ib.
Mr. Kimble on Idiopathic Hdyrophobia . . . . ib.
Dr. Petherbridge on Quinine in Intermittent and Remittent Fever . . 560
Dr. Flint on Quinine in Agues . . . . . ib.
Dr. Graves on Morbus Brightii. Is there such a Disease? . . ib.

PAGE
Dr. Gregory on Non-Identity of Smallpox and Cowpox . . . 560
Mr. Crisp on Gallstones . . . . . . 561
Dr. Patterson on Pseudo-Morbid Appearances of the Brain and its Envelopes . 562
Mr. Chambers on Perforation of the Stomach by a Worm . . . 563
Dr. Simpson on Leprosy and Leper Hospitals . . . . ib.

### SURGERY.

Prolapsus of the Uterus and anterior Wall of the Bladder . . . ib.

Dr. Scratchley on a Wound of the Axillary Artery . . . 564

Dr. Morris's Successful Operation for Prolapsus Uteri . . . ib.

Mr. Gay's Dissection of a Case of Spinal Curvature . . ib.

Mr. Blake's New Treatment of Aneurism . . . . ib.

Dr. Adair Laurie on the comparative merits of Amputation, &c. . . 565

Mr. Ewen's successful Case of Tracheotomy . . . . ib.

Mr. J. Toogood on Paraphymosis cured without Operation . . . ib.

Mr. Miller on Ligature of the Carotids . . . . ib.

Mr. Pugh on Closure of the Os Uteri . . . . . 566

Mr. Cooper on Strangulated Hernia treated with Opium . . . ib.

Dr. Browne's Case of successful Operation for Lateral Curvature . . ib.

Mr. Symes on Deformity from Rheumatism. Operation . . . ib.

Mr. Toogood on Propriety of Operating in Cases of Spreading Gangrene . 567

Mr. Duke on Hypertrophy of the Parotid Gland . . . ib.

### MIDWIFERY, AND DISEASES OF WOMEN AND CHILDREN.

Mr. Blenkinsop on Laceration of the Vagina, &c. . . . 568
Mr. Letheby on Kiestein as a Sign of Pregnancy . . . . ib.
Mr. Grantham on Fœtal Disease . . . . . . ib.
Dr. Grieve on a Knot on the Funis, causing Death of the Fœtus . . ib.
Dr. Tyler on Presentation of the upper extremities, and Spontaneous Evolution . ib.
Dr. Lockhart's Chinese Midwifery Case . . . . . 569
Dr. Duncan on Croup . . . . . . . ib.
Dr. Catlett on Ergot of Rye . . . . . . 570
Mr. J. Bell's Analysis of 265 Presentations in Labour . . . ib.
Dr. Stark on a New Substance, Gravidine, as a sign of Pregnancy . . 571
Dr. Montgomery on Cancerous Affections of the Uterus . . . ib.

### MEDICAL JURISPRUDENCE AND TOXICOLOGY.

Mr. J. Toogood on a Recovery after two drachms of arsenic were taken . 572
Dr. Guy on Static Lung Tests . . . . . . ib.
Mr. Barry on insufficiency of Albumen, &c. . . . . 574
Mr. Everest's Case of Poisoning with a minim and a half of Laudanum. . ib.

### ANIMAL CHEMISTRY AND PHARMACY.

Dr. Ure's Experiments on Asafœtida . . . . . 575

Mr. J. Mackay on Artificial Harrowgate Salts . . . . ib.

### MEDICAL STATISTICS.

Dr. C. Wilson on the Health of the Labouring Population of Kelso, &c. . 576

---

### Part Fourth.—Medical Intelligence.

New Bills of Mortality Tables for London . . . . 577

The Doctors *v.* the Assurance Societies . . . . . 578

Syrian Medical Association . . . . . . . 579

Medical Reform . . . . . . . . 580

Books received for Review . . . . . 581

Title, Index, &c.

